# Prevalence of methicillin-resistant *Staphylococcus aureus* and pattern of antimicrobial resistance in mastitis milk of cattle in Chitwan, Nepal

**DOI:** 10.1186/s12917-021-02942-6

**Published:** 2021-07-07

**Authors:** Asmita Shrestha, Rebanta Kumar Bhattarai, Himal Luitel, Surendra Karki, Hom Bahadur Basnet

**Affiliations:** 1 Department of livestock product and production management, Directorate of Agricultural Research, Lumle, Nepal; 2 Department of Veterinary Microbiology and Parasitology, Faculty of Animal Science, Veterinary Science and Fisheries, Agriculture and Forestry University, Rampur, Nepal; 3 Center for Biotechnology, Faculty of Agriculture, Agriculture and Forestry University, Rampur, Nepal; 4 Department of Epidemiology and Public Health, Himalayan College of Agricultural Sciences and Technology, Kathmandu, Nepal

**Keywords:** Antimicrobial resistance, Mastitis, MRSA, *mecA*, *S. aureus*

## Abstract

**Background:**

The threat of methicillin-resistant *Staphylococcus aureus* (MRSA) exists globally and has been listed as a priority pathogen by the World Health Organization. One of the sources of MRSA emergence is livestock and its products, often raised in poor husbandry conditions. There are limited studies in Nepal to understand the prevalence of MRSA in dairy animals and its antimicrobial resistance (AMR) profile. A cross-sectional study was conducted in Chitwan, one of the major milk-producing districts of Nepal, from February 2018 to September 2019 to estimate the prevalence of MRSA in milk samples and its AMR profile. The collected milk samples (*n* = 460) were screened using the California Mastitis Test (CMT) and positive samples were subjected to microbiological analysis to isolate and identify *S. aureus*. Polymerase Chain Reaction (PCR) was used to identify the presence of the *mecA* gene and screen for MRSA.

**Results:**

In total, 41.5% (191/460) of milk samples were positive in the CMT test. Out of 191 CMT positive milk samples, the biochemical tests showed that the prevalence of *S. aureus* was 15.2% (29/191). Among the 29 *S. aureus* isolates, 6.9% (2/29) were identified as MRSA based on the detection of a *mecA* gene. This indicates that that 1.05% (2/191) of mastitis milk samples had MRSA. The antibiotic sensitivity test showed that 75.9% (22/29) and 48.3% (14/29) *S. aureus* isolates were found to be sensitive to Cefazolin and Tetracycline respectively (48.3%), whereas 100% of the isolates were resistant to Ampicillin. In total 96.6% (28/29) of *S. aureus* isolates were multidrug-resistant (MDR).

**Conclusions:**

This study revealed a high prevalence of *S. aureus*-mediated subclinical mastitis in dairy herds in Chitwan, Nepal, with a small proportion of it being MRSA carrying a *mecA* gene. This *S. aureus,* CoNS, and MRSA contaminated milk poses a public health risk due to the presence of a phenotype that is resistant to very commonly used antibiotics. It is suggested that dairy herds be screened for subclinical mastitis and treatments for the animals be based on antibiotic susceptibility tests to reduce the prevalence of AMR. Furthermore, future studies should focus on the *Staphylococcus spp.* to explore the antibiotic resistance genes in addition to the *mecA* gene to ensure public health.

**Supplementary Information:**

The online version contains supplementary material available at 10.1186/s12917-021-02942-6.

## Background

*S. aureus* is an opportunistic organism that is found to colonize the skin and mucous membranes of 20–80% of the human population permanently and without symptoms [1]. Colonization and infection of livestock by *S. aureus*, as well as the exchange of virulence factors between human and livestock strains have been documented [2]. The symptoms associated with this bacterium range from simple skin infections to serious conditions like endocarditis and sepsis in humans. In livestock species, the bacteria cause wound infection, osteomyelitis, post-surgical abscess, and mastitis [3]. This shows that *S. aureus* lacks host specificity and causes a wide range of diseases. *S. aureus* is a diverse organism, and it has been evolving into a more developed and resistant form [4]. One of its variants, Methicillin-resistant *Staphylococcus aureus* (MRSA), also known as “resistant staph” or “superbug” is considered one of the major bacteria causing human infections in hospital and community settings. MRSA is listed as a high priority bacterium for further research and treatment by the World Health Organization [5]. Genetic mutation and resistance of MRSA to antibiotics commonly used in the field increases challenges to health workers [6]. *S. aureus* and MRSA were found to be the major causative agent of mastitis in Pokhara [7] and Bhaktapur [8], whereas Coagulase Negative Staphylococci (CoNS) was the predominant organism causing mastitis in Chitwan [9]. A study at Kathmandu Medical College has shown that 25.0% of MRSA isolates from humans are found resistant to Penicillin, Oxacillin, Cephalexin, and Erythromycin, which is similar to the animal isolate’s antibiotic resistance phenotype [10]. An in vitro drug sensitivity test has revealed the resistance ability of the mastitis pathogen, *S. aureus,* towards Ampicillin and Penicillin [11]. Thus, the above study has revealed the diverse resistance phenotype of *S. aureus*, particularly towards Ampicillin and Penicillin. In addition to this, the existence of a high occupational risk to the people viz. milkers, farmers, and veterinarians, having close contact with MRSA-infected cattle has also been mentioned [12]. The surveillance of such superbugs should be regularly conducted as milk is a daily consumed food and is supplied in a huge amount daily. The genetic background and antimicrobial resistance phenotype of *S. aureus* and MRSA from Chitwan dairy farms haven’t been studied yet. Therefore, a study was conducted to understand the prevalence and resistance characteristics of *S. aureus* and MRSA, and to provide help in the rational use of antibiotics.

## Results

### Prevalence of subclinical and serious mastitis infection in dairy cattle

At first, for determining the prevalence of MRSA in the milk samples of Chitwan, CMT positive milk samples were randomly collected. Among the CMT positive milk samples, as per the instruction of Bekuma and Galessm [13], the prevalence of subclinical and clinical mastitis were identified as 71.7% (*n* = 137/191) and 28.3% (*n* = 54/191), respectively. Overall prevalence of subclinical and clinical mastitis were 29.8% (137/460) and 11.7% (54/460), respectively. Out of 191 CMT positive milk samples cultured, the biochemical tests identified 29 milk samples (15.2%) with *S. aureus* and 30 (15.7%) with CoNS.

### Prevalence of MRSA

**Fig. 1:**
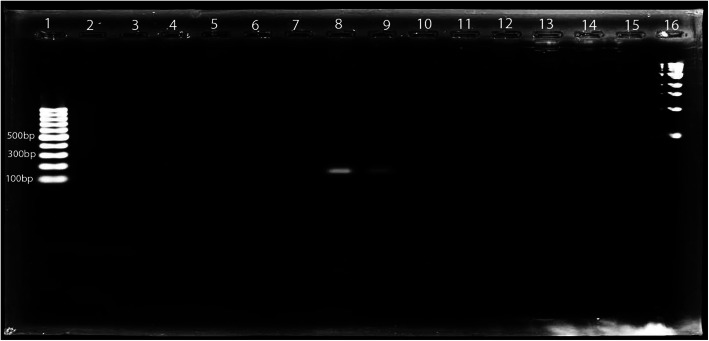
Visual of Gel through ultraviolet radiation showing *mecA* gene. Lane 1: 1 kb ladder; Lane 16: 100 bp ladder; Lane 2–15: isolates with positive isolates at 8th and 9th lane

The antibiotic sensitivity tests showed that 28 *S. aureus* isolates were resistant to Cefoxitin (30 μg) with a zone of inhibition measuring less than 21 mm. Thus, the prevalence of Oxacillin resistant *S. aureus* was 14.7% (28/191), but a *mecA* gene was detected in only two isolates (Fig. 1). The overall prevalence of MRSA on a genetic basis was 1.05% (2/191). Among the *S. aureus* isolates, 6.9% (2/29) were identified as MRSA on a molecular basis.

### Antibiogram profile of *S. aureus* isolates

Out of 11 antibiotics tested, 75.9% (22/29) *S. aureus* isolates were found to be the most sensitive to Cefazolin, while 51.7% (15/29) isolates were found to have an intermediate resistance to Erythromycin. Similarly, 100% (29/29) of the *S. aureus* isolate showed resistance to Ampicillin. A detailed description is provided in Table 1.

**Table 1:** Antibiogram profile of *S. aureus* and CoNS isolates

Antibiotic	Disc Potency (μg)	Sensitive		Intermediate		Resistance	
		%(no.)		%(no.)		%(no.)	
		*S. aureus*	CoNS	*S. aureus*	CoNS	*S. aureus*	CoNS
CIP	5	0 (0)	8.5 (3)	31.0 (9)	49.2 (15)	69.0 (20)	42.4 (13)
TE	30	48.3 (14)	52.5 (16)	31.0 (9)	30.5 (9)	20.6 (6)	17 (5)
TEI	30	10.3 (3)	11.5 (3)	0 (0)	0 (0)	89.7 (26)	88.1 (26)
AMP	10	0 (0)	0 (0)	0 (0)	0 (0)	100 (29)	100 (30)
CD	2	0 (0)	0 (0)	27.6 (8)	42.4 (13)	72.4 (21)	57.6 (17)
E	15	0 (0)	0 (0)	51.7 (15)	62.7 (19)	48.3 (14)	37.3 (11)
AK	30	3.5 (1)	6.8 (2)	10.3 (3)	20.3 (6)	86.2 (25)	72.9 (22)
GEN	10	6.9 (2)	20.3 (6)	0 (0)	0 (0)	93 (27)	79.7 (24)
CTX	30	6.9 (2)	5.1 (2)	48.3 (14)	45.8 (14)	44.8 (13)	49.2 (15)
CPM	30	10.3 (3)	20.3 (6)	20.7 (6)	27.1 (8)	69 (20)	52.5 (16)
CZ	30	75.9 (22)	78 (23)	6.5 (2)	8.5 (3)	17.2 (5)	13.6 (4)

*S. aureus = Staphylococcus aureus; CoNS = Coagulase Negative Staphylococcus spp.*

### Antibiogram profile of CoNS

Out of 11 antibiotics tested, 78.0% (23/30) of CoNS isolates were found to be the most sensitive to Cefazolin and 62.7% (19/30) of isolates had an intermediate response to Erythromycin. Similarly, 100% of the CoNS isolate showed resistance to Ampicillin. A detailed description is provided in Table 1.

### Resistance profile of *S. aureus* isolates

The resistance profile of *S. aureus* isolates showed 23 different phenotypes. Four isolates were resistant to at least seven groups of antibiotics, whereas seven isolates were resistant to six groups of antibiotics. Three isolates showed the same phenotypic character with a resistance pattern; CIP-GEN-AMP-CD-AK-CPM-CTX-TEI-E and a Multiple Antibiotic Resistance (MAR) index of 0.8. Eleven of the isolates had a MAR index of 0.5, and nine isolates were found to have a MAR index of 0.7. An additional Excel file is provided to explain this in more detail (see Additional file 1).

### Resistance profile of CoNS

The resistance profile of CoNS isolates showed 25 different phenotypes. An isolate was resistant to a maximum number of 10 antibiotics belonging to eight groups of antibiotics. Maximum isolates showed the same phenotypic character with a resistance pattern; GEN-AMP-AK-TEI and a MAR index of 0.4. In total, 23 number of CoNS isolates were found to be multidrug-resistant. Thirteen isolates were resistant to at least five groups of antibiotics. An additional Excel file is provided to explain this in more detail (see Additional file 2).

### Resistance profile of MRSA isolates

Out of 29 *S. aureus* isolates*,* only two Oxacillin resistant MRSA (OR-MRSA) were detected with a MAR index of 0.8 and 0.9. The two resistance patterns of MRSA were CIP-GEN-AMP-CD-AK-CPM-CTX-TEI-E, and CIP-TE-GEN-AMP-CD-AK-CPM-CTX-TEI-CZ-E. The other twenty-seven *S. aureus* isolates were Oxacillin Resistant but *mecA* negative (OR- MSSA).

### Species richness and MAR index of *S. aureus* and CoNS isolates

Out of 29 *S. aureus* isolates, 31.1% (9/29) were resistant to at least eight antibiotics tested, with a MAR index of 0.7, and eight isolates (27.6%) were resistant to at least six antibiotics with a MAR index of 0.5. Two isolates (6.9%) were resistant to 10 antibiotics (with a MAR index of 0.9). Out of 30 CoNS isolates, eight isolates were resistant to at least five antibiotics tested with a MAR index of 0.5. One isolate was resistant to 10 antibiotics with a MAR index of 0.9. Table 2 shows the number of isolates and their MAR indices.

**Table 2:** Species richness and MAR index of *S. aureus* and CoNS isolates

Total antibiotics used	No of Antibiotic-resistant (species richness)	MAR index	No of isolates resistant	
			*S. aureus* [14]	CoNS [15]
11	1	0.1	0	1
	2	0.2	1	2
	3	0.3	1	7
	4	0.4	0	0
	5	0.5	3	8
	6	0.5	8	6
	7	0.6	1	3
	8	0.7	9	1
	9	0.8	4	1
	10	0.9	2	1

The mean MAR index of *S. aureus* is significantly higher than that of CoNS isolates (t_57_ = 3.168_;_ *p* < 0.05)

*MAR* Multiple Antibiotic resistance, *S. aureus Staphylococcus aureus, CoNS* Coagulase-negative *Staphylococcus spp*

### MDR phenotype of *S. aureus* and CoNS isolates

Out of 29 *S. aureus* isolates tested against seven groups of antibiotics, 28 (96.6%) isolates were resistant to antibiotics belonging to more than three groups of antibiotics tested and were classified as MDR isolates.

Out of 30 CoNS isolates tested against seven groups of antibiotics, 27 isolates (90.0%) were resistant to antibiotics belonging to more than three groups of antibiotics tested and were classified as MDR isolates.

## Discussion

This cross-sectional study conducted in dairy farms in Chitwan district, one of the major milk-producing districts of Nepal, showed that the prevalence of sub-clinical or clinical mastitis was 41.5% (191/460). *S. aureus* (15.2%, 29/191 samples) and CoNS (15.7%, 30/191 samples) were identified as significant bacteria causing mastitis. In total, 1.05% (2/191) of isolates from mastitis milk and 6.9% (2/29) of *S. aureus* isolates were MRSA that carried a *mecA* gene. The majority of these isolates were found to be multidrug-resistant.

*S. aureus* was identified as one of the major bacteria causing mastitis in dairy cattle in this study. This finding is consistent with previous studies conducted in Nepal, which had also shown *S. aureus* as one of the major bacterial pathogens causing clinical and sub-clinical mastitis [16, 17]. However, these previous studies did not evaluate *S. aureus* isolates at the genetic level.

In this study, two mastitis milk samples (1.05%, 2/119) and 6.9% (2/29) of *S. aureus* samples were found to be carrying a *mecA* gene, thereby confirming them as MRSA. The presence of MRSA in milk from dairy cattle poses a risk to public health if preventive measures are not applied. In a similar study conducted in Tamil Nadu India, MRSA was detected in 3.0% (12/409) of the mastitis milk samples, which was slightly higher than in this study [17]. Likewise, in a study conducted in Northwestern China in 2014, a high prevalence (56.5%, 121/214) of *S. aureus* was determined, but only one isolate (0.46%) was determined to be MRSA, which was slightly lower than what was found in our study [18]. Similarly, seven *S. aureus* isolates were resistant to Oxacillin, but did not carry a *mecA* gene, which supports the findings of this study [18]. However, in a study conducted in four provinces of China, 47.6%(49/103) of *S. aureus* isolates from milk samples were identified as MRSA with the *mecA* gene [19], which is significantly higher than isolated in this study and other studies in the region [19, 20]. MRSA carrying the *mecA* gene has been found in varying proportions in different parts of the world, for example, 42.9% (*n* = 64) in Tigray, Ethiopia [20], 35.7% (*n* = 84) in Egypt [21], 9% (*n* = 728) in Karnataka, India [22], 0.8% (*n* = 363) in bulk milk tanks in England and Wales [23].

The AMR patterns of *S. aureus* showed that they are highly resistant to a majority of the commonly available antibiotics on the market. For example, *S. aureus* isolates were 100% resistant to Ampicillin and, to some degree, also resistant to Ciprofloxacin, Gentamicin, Teicoplanin, and Cefemine. The *S. aureus* and CoNS isolates were found to be relatively sensitive to Tetracycline and Cefazolin. In a study conducted in Ethiopia, isolates were highly resistant to Ampicillin, but were sensitive to Amikacin, Gentamycin, and Tetracycline [20]. A high level of MDR *S. aureus* was also found in several other studies [17, 18, 20–25]. Based on the antibiogram profile of *S. aureus* and CoNS, fourth-generation Cephalosporin group of antibiotic, Cefazolin, and tetracycline are the drugs-of-choice against *S. aureus* in mastitis. Similar results were obtained by Memon [26] in China. More than 25.0% of MRSA isolates were resistant to Penicillin, Oxacillin, Cephalexin, Co-trimoxazole, and Erythromycin [10]. The high level of AMR, including MDR, indicates irrational use of antibiotics is practiced in the dairy farms of Chitwan.

A limitation of this study is that only a *mecA* gene was evaluated to determine MRSA, while another gene named *bla*Z is also a specific gene for MRSA and responsible for resistance to β-lactam antibiotics [27]. Thus, the prevalence of MRSA determined in this study might be an underestimation. Furthermore, alternative genes other than *mecA,* like *mecA*_*LGA251*_ and *mecC* could be present in a microorganism leading to the MDR characteristic, which is supported by the study of Denmark [28]. Besides, risk factors related to the presence of MRSA were not examined and evaluated.

## Conclusions

This study has revealed that *S. aureus*-mediated subclinical mastitis is widely prevalent in Chitwan dairy herds. Among the mastitis milk, 15.2% (29/191) of the milk samples were positive for *S. aureus*. Among the 29 samples tested positive for *S. aureus*, two samples were confirmed carrying a *mecA* gene, indicating the 6.9% (2/29) prevalence of a *mecA* gene among the *S. aureus* isolates in the Chitwan district. The antibiotic sensitivity pattern revealed that *S. aureus* was sensitive to Cefazolin and Tetracycline, but resistant to Ampicillin, Amikacin, Teicoplanin, and Gentamycin. It was found that 96.6% (28/29) of *S. aureus* and 90.0% (27/30) of CoNS isolates were multidrug-resistant isolates. As MRSA is also an important human pathogen, circulation of MRSA bacteria in dairy cattle poses a risk to public health.

## Methods

This cross-sectional study was conducted from February 2018 to September 2019 in the Chitwan district, located in the Bagmati Province of Nepal, on 71,864 dairy cows [29]. A total of 23 farms were selected from the pocket areas: Rampur, Mangalpur, Gitanagar, Mahendrachowk, Divyanagar, and Ratnanagar, by lottery system of random sampling. Cows within the farms were selected by the same method.

The sample size formula provided by Daniel [30] was used, which is

$$ n=\frac{Z^2p\left(1-p\right)}{d^2} $$

Where,

Z: Z statistic for a level of confidence. (For the level of confidence of 95.0%, which is conventional, Z value is 1.96).

P: expected prevalence or proportion. P is considered 0.11, the herd-level prevalence of MRSA in cattle of Pokhara [7].

d: precision (d is considered 0.05 to produce good precision and smaller error of estimate).

Therefore, sample size (*n*) = 150.4 from the total dairy cattle population of 71,864 in Chitwan.

The milk samples were collected according to the protocol of the National Mastitis Council [14] from the healthy teats of dairy cattle. After a quarter of a cattle was washed with tap water and dried, the teat end was swabbed with cotton soaked in 70.0% ethyl alcohol. Milk with a change in color and consistency was discarded. In a large herd, one cow was selected at random from the group of five cows. In total, 460 udder quarters from 115 cows were subjected to the California Mastitis Test (CMT) and the scores were given as per Bekuma and Galessm [13]. A score of “0” was regarded as a healthy udder quarter; score “1” as subclinical mastitis; score “2” and “3” as serious mastitis. A total of 191 udder quarters were CMT positive with scores 1, 2, and 3. Thus, the sample size of this study was 191. Approximately 3 ml of milk was collected aseptically from CMT positive quarter into the sterile tube after discarding the first three milking streams. Those samples were transported in a thermo-cool box to the Laboratory of Veterinary Microbiology, FAVF, Rampur.

The milk samples in the thermo-cool box were thawed before pre-enrichment. One milliliter of the sample was dispensed into a test tube containing nine milliliters of sterile peptone water (M028, HiMedia, India) to make a ratio of 1:9 and was incubated at 37 °C for 24 h. A loopful of the enriched peptone water was taken by a sterile inoculum loop and streaked onto Mannitol Salt Agar (MSA) (M118, Himedia, India). The streaked plate was incubated overnight at 37 °C. The suspected golden-yellow colonies on MSA were sub-cultured on nutrient agar to gain a pure colony of *Staphylococcus* and incubated overnight at 37 °C. A gram staining test, catalase test, oxidase test, coagulase test, and hemolysis test were performed for confirmation of *S. aureus*. An antibiotic sensitivity test for *Staphylococcus spp.* was performed by the Kirby-Bauer disk diffusion method on Mueller-Hinton agar [15]. An inoculum was prepared by emulsifying pure colonies in sterile normal saline (0.85%) in Eppendorf tubes with the turbidity adjusted to 0.5 McFarland standards equivalent to 1.0 × 10^8^ cfu/mL. The inoculum thus prepared was uniformly streaked on Mueller Hinton agar plates using sterile swabs and left for a minute prior to introduction of the antibiotics. Antibiotics commonly used in the field were selected, named as Ampicillin(AMP), Gentamycin(GEN), Erythromycin(E), Ciprofloxacin(CIP), Tetracycline(TE), Clindamycin(CD), Teicoplanin(TEI), Amikacin(AK), Cefotaxime(CTX), Cefepime(CPM), and Cefazoline(CZ). The plates were incubated at 37 °C for 24 h, and the diameters of the zones of inhibition were measured, and results interpreted according to HiMedia interpretative chart [31].

### Detection of Methicillin-resistant *S. aureus*

#### Cefoxitin-based methods

An antibiotic sensitivity test for *S. aureus* was performed with the Kirby-Bauer disk diffusion method on Mueller-Hinton agar [15] against the Cefoxitin disc of 30 μg potency. After incubation, the diameter of the zone of inhibition was recorded to the nearest millimeter of each disc by Hi Antibiotic Zone Scale (HiMedia, India) and then classified as sensitive, intermediate, and resistant according to the manufacturer’s interpretative chart [31].

#### Polymerase chain reaction

The DNA of *S. aureus* was extracted using DNeasy Blood and Tissue Kit (Qiagen, German Product) and stored in a sterile Eppendorf tube. The concentration and purity of DNA extracted from *S. aureus* were quantified by spectrophotometer at 260 nm and 280 nm using a nano spectrophotometer (Quawell, UV-Vis Spectrophotometer, Q5000 V6.0.2). For identification and confirmation of the MRSA, the “*mecA*” gene of 124 bp was used as a molecular marker with an oligonucleotide sequence of forward and reverse primer GAATGCAGAAAGACCAAAGCA and TTTGGAACGATGCCTATCTCA, respectively [32].

The polymerase chain reaction (PCR) was performed using *S. aureus* specific methicillin-resistant gene *mecA* for the identification of MRSA. The reaction mixture consisted of 1 μl genomic DNA, 10 μl Hot Start Taq 2X master mix (Biolabsline), 1 μl of each primer, and the final volume was adjusted to 20 μl by adding nuclease-free water. After mixing all the components in PCR tubes, DNA was added and the tubes were placed in the Bio-Rad T100™ Thermal Cycler (Bio-Rad, USA) to preheat at 95 °C. A total of 40 PCR cycles were run under the following conditions: initial DNA denaturation at 95 °C for 5 min, denaturation 95 °C for 1 min, primer annealing at 52 °C for 45 s, and DNA extension at 72 °C for 1 min. After the final cycle, the reaction was terminated by maintaining at 72 °C for 5 min. The PCR products were stored in the cycler at 4 °C until they were collected.

The amplified PCR products were resolved by electrophoresis in 1.5% agarose gel. The agarose powder (0.9 g) was added to 60 ml of 1% Tris-Boric acid –EDTA (TBE) along with 6 μl Syber safe (S33102, Invitrogen) to prepare 1.5% agarose gel. The gel was put in an electrophoresis tank (MultiSUB Electrophoresis Systems; Nano PAC-300P, Cleaver Scientific) containing 450 ml of 1.0% TBE buffer. The first and last wells of the gel were loaded with 6 μl of 100 bp DNA ladder (100 μg/ml) and 1 kb DNA ladder (500 μg/ml), respectively. The rest of the wells were loaded with 6 μl of a mixture prepared by adding 2 μl of 6X DNA loading dye (Thermo Scientific) to 6 μl of sample DNA. The gel electrophoresis was then set and run at 85 V, 90A for 70 mins, and visualized under UV trans-illuminator (Platinum Q9, Uvitech Cambridge).

#### Data analysis

The data were recorded and maintained in Microsoft Excel 2007. A prevalence percentage was calculated by dividing the number of positive samples for the given category by the total samples tested within that category. The prevalence formula was applied for determining prevalence percentage of mastitis, *S. aureus*, CoNS, and MRSA. The AMR patterns, resistance, intermediate, and sensitivity were calculated using the CLSI guideline using the cut-off as provided in the brochure of the manufacturer (HiMedia, India).

## Supplementary Information

**Additional file 1 **Resistance profile of *S. aureus* isolates. Based on antibiotic susceptibility test of *S. aureus* against commonly used antibiotics, resistance profile of each isolates were determined. Altogether 23 different resistance profiles of isolates were determined.

**Additional file 2.** Resistance profile of CoNS isolates. Based on antibiotic susceptibility test of CoNS against commonly used antibiotics, resistance profile of each isolates were determined. Altogether, 25 different resistance profiles of isolates were determined.

## Data Availability

Raw data in this study were generated through CMT test, AST test and biochemical test, which are available from the corresponding author on reasonable request.

## Abbreviations

Α: Alpha
A: Ampere
AD: After Death
AK: Amikacin
AMP: Ampicillin
AST: Antibiotics Sensitivity Testing
Bp: Base pair
Β: Beta
CD: Clindamycin
CDC: Centers for Disease Control and Prevention
CIP: Ciprofloxacin
Cl: Chlorine
CLSI: Clinical and Laboratory Standards Institute
CM: Clinical Mastitis
CMT: California Mastitis Test
CoNS: Coagulase Negative *Staphylococcus spp*
CPM: Cefepime
CTX: Cefotaxime
CV: Crystal Violet
CX: Cefoxitin
CZ: Cefazolin
E: Erythromycin
GEN: Gentamicin
HA: Hospital Associated
H_2_O_2_: Hydrogen Peroxide
I: Intermediate
Kb: Kilobyte
LA: Livestock Associated
MAR: Multiple Antibiotic Resistance
MDR: Multidrug Resistant
MHA: Mueller Hinton Agar
MRSA: Methicillin Resistant *S. aureus*
MSA: Mannitol Salt Agar
NA: Nutrient Agar
NB: Nutrient Broth
O: Oxacillin
OR: Oxacillin Resistant
OS: Oxacillin Sensitive
pH: the negative logarithm of H^+^ Concentration; Acidity
PBP: Penicillin Binding Protein
PCR: Polymerase Chain Reaction
R: Resistant
S: Sensitive
SCC: *Staphylococcus* Cassette Chromosome
SCM: Subclinical Mastitis
SE: *Staphylococcus* Enterotoxin
spp.: Species
TE: Tetracycline
Tei: Teicoplanin
